# Baseline correction of phase-contrast images in congenital Cardiac Magnetic Resonance Imaging

**DOI:** 10.1186/1532-429X-11-S1-P135

**Published:** 2009-01-28

**Authors:** Brian J Holland, Beth F Printz, Wyman W Lai

**Affiliations:** Children's Hospital of New York, New York, NY USA

**Keywords:** Congenital Heart Disease, Aortic Regurgitation, Main Pulmonary Artery, Leave Pulmonary Artery, Pulmonary Regurgitation

## Introduction

Phase-contrast flow measurements have been shown to accurately quantify blood flow in subjects with structurally normal hearts and those with congenital heart disease (CHD). There are a number of potential sources of error, however, in phase-contrast CMR flow measurements, including phase offset errors due to local non-compensated eddy currents. Wolff and colleagues recently reported on the clinical application of phantom correction of phase offset errors in adult volunteers with structurally normal hearts [1]. The effect of phantom correction on phase-contrast flow measurements in patients with known or suspected heart disease has not previously been reported.

## Purpose

To assess whether phantom correction significantly changed flow measurements by phase-contrast imaging in patients referred to a congenital CMR program.

## Methods

The clinical congenital cardiac magnetic resonance imaging database at a single institution was searched for patients who had examinations performed with phase contrast images using phantom correction. Examinations were performed on a GE Signa HDx 1.5 T scanner using commercially available coils (GE Healthcare, Milwaukee, Wisconsin). Phase-contrast images were acquired perpendicular to the vessel of interest using orthogonal long-axis views of the vessel. Breathe-through images were obtained using the commercially resident FastCine PC pulse sequence with the Venc determined by clinical parameters. The phase-contrast sequences were each repeated on a stationary fluid phantom with an ECG simulator to establish a baseline of zero velocity. Based on the clinical protocol, phase-contrast images of flow were obtained in the ascending aorta (AAO), main pulmonary artery (MPA), right pulmonary artery (RPA), and/or left pulmonary artery (LPA) and analyzed with and without phantom correction using GE ReportCard software version 3.6. The ratio of pulmonary to systemic flow (Qp/Qs), percent flow to the RPA (QpR fraction), pulmonary regurgitation fraction (PR), and aortic regurgitation (AR) fraction were also calculated with and without phantom correction. Clinically significant changes in flow measurements with phantom correction were defined prior to analysis: a change in MPA or AAo flow ≥ 0.5 L/min/m^2^, change in RPA or LPA flow ≥ 0.25 L/min/m^2^, change in Qp/Qs ≥ 0.4, change in QpR fraction ≥ 10%, and change in PR or AR fraction ≥ 10%. Marked changes in flow measurements were defined as double the amount of clinically significant change in each category – for example, a change in MPA flow ≥ 1.0 L/min/m^2^ or PR fraction ≥ 20%.

## Results

From May 2008 to September 2008, 89 patients (median age 17.5 years, range 0.3 to 58.7 years) were identified who had clinical CMR examinations using phase-contrast images with phantom correction. The patients were referred with the following diagnoses: 25 with tetralogy of Fallot (repaired), 17 miscellaneous, 12 other conotruncal diagnoses, 11 single ventricle, 9 shunt lesions, 9 cardiomyopathy/myocarditis, and 6 aortic coarctation. The number and percent of patients with clinically significant or marked changes in the phase-contrast measurements are listed in Table 1.

**Table 1:** Phantom correction of phase-contrast measurements

Variable	N	Clinically Significant Change	MarkedChange
MPA flow (L/min/m2)	67	29 (43%)	11 (16%)
AAO flow (L/min/m2)	85	32 (38%)	16 (19%)
RPA flow (L/min/m2)	38	9 (24%)	5 (13%)
LPA flow (L/min/m2)	33	17 (52%)	11 (33%)
Qp/Qs	64	12 (19%)	5 (8%)
PR fraction (%)	28	7 (25%)	0 (0%)
AR fraction (%)	8	3 (38%)	0 (0%)
QpR fraction (%)	30	8 (27%)	3 (10%)

## Conclusion

Phantom correction in our study population resulted in clinically significant changes in 19% to 52% of phase-contrast measurements in patients with known or suspected heart disease. There were marked changes in up to 33% of phase-contrast measurements. Clinically significant or marked changes were generally more common in measurements of output (L/min/m^2^) as compared to regurgitant fractions or flow ratios. The potential effect of phantom correction of phase-contrast images may be important in clinical decision making for patients with congenital heart disease. The magnitude of change should be assessed on other CMR platforms and in additional patient groups.
